# Indirect effects of cytomegalovirus infection: Implications for vaccine development

**DOI:** 10.1002/rmv.2405

**Published:** 2022-11-15

**Authors:** Philip Moseley, Paul Klenerman, Seilesh Kadambari

**Affiliations:** ^1^ Department of Paediatrics Horton General Hospital Oxford University Hospitals Banbury UK; ^2^ Translational Gastroenterology Unit University of Oxford Oxford UK; ^3^ Nuffield Department of Medicine University of Oxford Oxford UK; ^4^ Department of Paediatrics Oxford Vaccine Group University of Oxford Oxford UK; ^5^ NIHR Oxford Biomedical Research Centre Oxford UK; ^6^ Department of Paediatric Infectious Diseases Great Ormond Street Hospital for Children NHS Foundation Trust London UK; ^7^ Great Ormond Street Institute of Child Health University College London London UK

## Abstract

Development of a cytomegalovirus (CMV) vaccine is a high priority due to its significant global impact—contributing to mortality in immunosuppressed individuals, neurodevelopmental delay in infected neonates and non‐genetic sensorineural hearing loss. The impact of CMV on the general population has been less well studied; however, a wide range of evidence indicates that CMV may increase the risk of atherosclerosis, cancer, immunosenescence, and progression of tuberculosis (TB) and human immunodeficiency virus. Due to the high seroprevalence of CMV worldwide, any modulation of risk by CMV is likely to have a significant impact on the epidemiology of these diseases. This review will evaluate how CMV may cause morbidity and mortality outside of the neonatal and immunosuppressed populations and consider the potential impact of a CMV vaccine on these outcomes.

**Abbreviations**
CMV: cytomegalovirus
CRP: C‐reactive protein
CVD: cardiovascular disease
EOD: end organ damage
HCV: hepatitis C virus
HIC: high income country
HIV: human immunodeficiency virus
LMIC: low middle income country
PD‐1: programed death receptor 1
TAM: tumour associated macrophage
TB: tuberculosis
TCR: T‐cell receptor
Th1: Type 1 T‐helper cell

## 1 INTRODUCTION

Cytomegalovirus (CMV) is the most common non‐genetic cause of sensorineural hearing loss in neonates and a leading cause of neurodevelopmental delay.^1^ In the immunocompromised host, CMV reactivation results in vasculopathy, graft rejection and severe end organ damage.^2^, ^3^ Subsequently, the development of a vaccine against CMV has been cited as a high priority for the last 2 decades including by the National Institute of Health.^4^

CMV has been associated with several distinct disease processes– atherosclerosis,^5^, ^6^, ^7^, ^8^ cancer^9^ and impaired immune response to vaccination.^10^, ^11^, ^12^ Additionally, CMV is suspected to enhance the pathogenicity of human immunodeficiency virus (HIV) and tuberculosis (TB).^13^, ^14^, ^15^, ^16^ Animal studies and large population based epidemiological studies suggest that CMV may cause disease through mechanisms such as CD8+ memory T‐cell inflation^17^; immunosenescence and immunomodulation.^18^, ^19^, ^20^ In addition, reactivation of CMV in local tissues may result in lytic replication and cause direct damage to tissues. However, current evidence has only demonstrated an association rather than causal role in these disease processes.

The primary objectives of developing a vaccine against CMV have been to eliminate congenital infection and to reduce morbidity and mortality in highly immunosuppressed individuals. However, this review presents evidence that CMV may also contribute to the development of vascular, infectious, and oncological conditions in the general population. Globally, CMV seroprevalence is estimated to be between 60% and 90%^21^ and therefore if there is any modulation in risk by CMV, vaccination is likely to have a significant impact on the incidence of these diseases. We used PubMed to perform a literature review of the epidemiology and potential pathogenic mechanisms of CMV using search terms ‘CMV’ or ‘cytomegalovirus’ or ‘human cytomegalovirus’ in combination with ‘atherosclerosis’, ‘cancer’, ‘HIV’, ‘TB’ or ‘vaccination’. We included these themes as they represent the most studied areas of CMV in the general population, and areas where there would be the greatest implications if a causal interaction did exist. We performed a separate ClinicalTrials.gov search for CMV vaccination trials. In this review, we focus on the potential indirect effects of CMV in the general population, and methods to evaluate the effect of vaccination on associated disease development during and after vaccine trials.

### 1.1 CMV in immune development

CMV seropositivity becomes increasingly prevalent with age. In low‐middle income country (LMIC) settings, the age of acquisition is typically younger—studies in the Gambia and Uganda demonstrated 86%–95% seroprevalence at 12 months of age compared to 15% in UK 1–4‐year‐olds.^22^, ^23^ Even in high income settings, the seroprevalence eventually reaches 60%–80% by 65 years of age.^22^, ^23^, ^24^ The age of CMV acquisition and the role of co‐infection with other viruses are factors that may influence how CMV interacts with the host immune system. The human virome is an emerging concept in virology, and similar to the microbiome, the interactions between host and virus are likely to shape immune development and response.^25^

CMV is a major determinant of variation in the immune system between individuals^26^ and therefore likely exerts an important effect on its development. CMV‐specific CD8+ effector memory T‐cells dominate the immune repertoire of elderly patients who are seropositive for CMV.^17^ This has been proposed due to chronic reactivation and low‐level presentation of antigens to CMV‐specific T‐cells leading to memory T‐cell inflation.^27^ In healthy monozygotic twins who are discordant for CMV seropositivity, the number of effector CD8 T‐cells and gamma‐delta T cells are poorly correlated.^26^ Similarly, there are significantly different plasma levels of IL‐10 and IL‐7 level between CMV seropositivity discordant twins, however this study did not adjust for any confounding factors that may explain this discrepancy.^26^ Overall, evidence indicates that CMV is strongly associated with phenotypical differences between individual immune systems. With increasing age, there is a diminishing impact of genetic factors in determining the proportion of cell populations, cytokines and signalling molecules—this may reflect an increasing role of microbial influences, of which CMV is a likely a major contributor.

CMV is linked to profound changes in various cellular subsets, including reduction in naïve cell populations, and presence of cellular phenotypes and functions.^28^ Overall, it appears that with age the T‐cell compartment becomes increasing dominated by CMV‐specific CD8+ T‐cells with a corresponding reduction in the naïve T‐cell population. In CMV seropositive experimental mice models naïve CD8+ T‐cells are reduced and a similarly reduced naïve cell population appears to contribute to an overall mortality risk profile.^29^, ^30^ CMV‐specific CD8+ cells typically re‐express CD45RA alongside a reduction in co‐stimulation markers such as CD28 and CD27.^20^ The CD45RA isoform becomes increasingly expressed during chronic CMV infection and is associated with expansion of low‐avidity cells. CD45RA, the longer isoform of CD45, is typically expressed on naïve T‐cells and impacts on the T‐cell receptor (TCR) signalling pathway through altering the interaction of the TCR with CMV antigens. Interestingly, these CMV‐specific T‐cells express few markers of T‐cell exhaustion such as programed cell death protein 1 (PD‐1), in contrast to T‐cells seen in chronic infection secondary to HIV and hepatitis C virus (HCV).^28^ This may be related to the low‐level of chronic exposure of antigens in CMV latent infection compared to the high level of damage and replication seen in HIV and HCV.

CD8+ CD28− CD57+ T‐cells is a specific subset which has been found to be elevated in cohorts of CMV seropositive patients compared to controls.^31^ These cells represent a highly differentiated phenotype with limited proliferative ability. CD57 is a surface sulphated glycan carbohydrate that has been found to be a marker of chronic immune activation in humans often in association with suppression of CD28. Studies suggest that this cell population can remain highly polyfunctional and maintain ability to kill pathogens ex vivo.^31^ Nevertheless, several studies have associated expansion of this T‐cell subset with increased mortality and morbidity.^32^, ^33^ The apparent contradiction of these findings may suggest that these cells are not directly pathogenic and may represent a proxy of chronic immune dysfunction and inflammation induced by CMV. Alternatively, these polyfunctional cells may be directly pathogenic through generation of excessive inflammation and bystander host damage and thus contributing to ‘inflamm‐ageing’.

Studying the relationships between age of acquisition and outcomes at different ages has been challenging due to the inherent difficulties in conducting long‐term observational studies spanning decades and the inability to date when primary infection occurred.

## 2 ATHEROSCLEROSIS

Many epidemiological studies have suggested an association between CMV seropositivity and cardiovascular mortality risk^5^, ^8^, ^34^, ^35^, ^36^; however, distinguishing whether this is a confounding effect of co‐variants such as socioeconomic status, country of origin and smoking status, makes establishing causality challenging. A large longitudinal study of 14,000 subjects in the USA recruited from the National Health and Nutrition Examination Survey (NHANES) III (1988–1994) showed that CMV seropositivity at initial sampling (age ranging from 25 to 90 years old) was associated with increased cardiovascular and all‐cause mortality after 2 decades of follow up^35^ (Table 1). The effect on all‐cause mortality (hazard ratio [HR] 1.19, 95% CI: 1.01–1.41) remained significant after adjusting for confounders including diabetes, age and gender; however, the effect on cardiovascular disease (CVD) mortality disappeared after adjustment.^35^ A nested USA case‐cohort of 726 participants reported that patients with the highest 20% of CMV immunoglobulin G (IgG) titres had an increased rate of CVD, with a HR of 1.76 (95% CI: 1–3.11), as compared to patients with the lowest 20% of CMV IgG titres.^8^ Conversely, in a 13,090 participant prospective study based in the UK which included 2514 deaths, there was no significant association between CVD mortality and CMV IgG seropositivity over an average 14.5 years follow up.^34^

**TABLE 1:** Summary of the key studies evaluating the indirect effects of cytomegalovirus (CMV)

Effect	Trial/study	Population	Exposures and outcomes measured	Results
Increased CVD risk/overall mortality	OCTA/NONA^32^, ^37^	Longitudinal	At several time points:	Concept of ‘immune risk profile’ (IRP)
		Swedish octogenarians, starting in 1989	CD4/CD8 ratio	
		101 participants originally	Lymphocyte subsets	High IRP patients has highest rate of mortality and CVD risk
			T‐cell responsiveness	
			CMV seropositivity	IRP variables include CMV seropositivity, CD4/CD8 inversion, CMV‐specific T cells
			CVD mortality/Overall mortality at 10 years follow up	
	EPIC^34^	30,000 men and women aged 40–79 years at baseline between 1993 and 1998 from 35 participating general practices in Norfolk, UK	CMV IgG level	Trend towards increase in CVD mortality in high IgG titre (HR 1.24). Non‐significant
			BMI/Socioeconomic data	
			Labs including CRP/lipids
			CVD mortality	High CMV IgG associated with all‐cause mortality when controlling for many confounders (HR 1.23)
			Cancer mortality	Significant associated between non‐cancer non‐CVD increased mortality (HR 1.35)
			Overall mortality	
	ARIC^8^	Nested case‐cohort, 45–64 years old, 726 participants, US communities in 4 states	CMV IgG level	High CMV IgG (top 20%) associated with increases coronary heart disease (HR 1.76) after adjustment for confounders
			HSV antibodies	
			Labs including lipids. No CRP	
			BMI/Socioeconomic data	
			Coronary heart disease (including MI deaths) at 5 years follow up	
	NHANES III^35^	US population study of 14,153 participants aged >25 years old; samples taken between 1988 and 1994	CMV seropositivity	CMV seropositivity associated overall mortality after confounders controlled (HR 1.19)
			Socioeconomic data	
			Lab data including CRP/lipids	High CRP and CMV seropositivity associated with CVD death (HR 1.67)
			CVD mortality and overall mortality measured in 2006	
	Sacramento area Latino study on ageing^38^	A longitudinal population‐based study of 1459 older Latinos (aged 60–101 years) in California, US followed in 1998–2008	CMV immunoglobulin G (IgG), tumour necrosis factor, and interleukin‐6	High CMV Ig (top 25% vs. lowest 25%) significantly increased CVD death (HR 1.35) after adjustment
			CVD mortality	High CMV Ig (top 25% vs. lowest 25%) significantly increased overall mortality risk (HR 1.43) after adjustment
				TNF, IL‐6 contributes to CMV associated risk
Increased cancer risk—glioma	CMV and glioma prognosis metanalysis^39^	7 studies identified up to 2019. Includes around 250 patients	CMV seropositivity	No association between CMV seropositivity and glioma prognosis
			CMV IgG levels	
			CMV IEA staining on histology samples	Increased CMV IEA staining on histology worsens progression free survival (HR 1.46)
			Progression free survival	
			Overall mortality	
	Detection of CMV in gliomas^40^	125 histological samples of GMB in the US	CMV IE1/2 detection via in situ hybridisation, PCR, immunohistochemistry in a combination of fresh‐frozen tissue samples and formalin‐fixed paraffin‐embedded	No detection of CMV protein or transcripts in any samples
	Detection of CMV in gliomas and peripheral blood^41^	45 cases of GBM diagnosed histologically in US	CMV DNA in peripheral blood	93% of GBM samples had CMV proteins stain positive
			CMV DNA in GBM histology	
			CMV IEA staining in GBM histology	80% of patients with positive CMV proteins stain on GBM histology had peripheral blood CMV DNA detected
Interaction with TB/HIV	General population cohort (Uganda)^42^	Rural Uganda, 25 patients with active TB; 256 controls with no active TB. Mean age 26	CMV IgG levels	Individuals with medium CMV IgG OR 2.8 to have active tuberculosis disease (*P* = 0.055), and those with high CMV IgG OR 3.4 to have active tuberculosis disease (*P* = 0.007)
			EBV/HSV/HIV antibodies	
			Serum cytokine levels	
			TB sputum AFB	
	MVA85A TB vaccine trial cohort (South Africa)^43^	Prospective South African cohort study. Part of vaccine study. 49 infants TB disease at first 2 years of life, versus 129 healthy matched controls	CMV IgG levels;	CMV+ 48.8% risk versus CMV− 25% risk of active TB at 2 years (*p* = 0.043)
			CMV‐stimulated IFN‐γ response	
			PBMC transcriptome analysis	
	NCT00953927		TB sputum AFB	Transcriptional activated T cells, type I IFN responses, and NK cells in infants up to 3 years prior to detection of TB
			Time to TB diagnosis	
Effect on response to vaccination	Polio vaccine response in infants^44^	369 CMV seropositive versus 75 CMV seronegative Zambian infants at 18 months of age	CMV seropositivity	No difference in antibody titres at 18 months of age between CMV serostatus group
			HIV status	
			HIV exposure during pregnancy	
		Part of randomised nutritional study	Socioeconomics factors	
			Breastfeeding	
			Polio antibody titre	
	Measles vaccination in infants^45^	Cohort study, 132 Gambian Newborns	CMV seropositivity	1 week post‐vaccination: Reduced IFN‐y response to measles in CMV+ ve; no other differences
			CMV DNA urine	
			Lymphocyte subsets	
			CD4+ IFN‐y production to measles protein	4‐month post‐vaccination: No difference in IFN‐y response to measles; no difference in cytokine production
			Measles antibody production: Inhibition of haemagglutinin protein assay	
	Influenza in adults^11^, ^12^	Part of safety study for seasonal flu vaccine	CMV seropositivity	Derhovanessian: >60 year old, CMV seropositivity was associated with reduction in influenza antibody production (88% vs. 44%, *p* = 0.033)
		54 subjects from Antwerp, Belgium. Age >18	Lymphocyte subsets	Frasca: In young (>18) and elderly (>60), CMV seropositivity was associated with reduction influenza Ab production
			Haemagglutinin inhibition assay	

Abbreviations: AFB, acid fast bacilli; BMI, body mass index; EIA, enzyme immunoassay; GBM, glioblastoma; HR, hazard ratio; HSV, herpes simplex virus; IEA, immediate eary antigen; IFN, interferon; MI, myocardial infarction; NK cell, natural killer cell; PBMC, peripheral blood mononuclear cell; PCR, polymerase chain reaction; TNF, tumour necrosis factor.

Evidence for the contribution of CMV to the development of atherosclerosis is derived from several sources. Firstly, antiviral therapy reduces the rate of transplant graft atherosclerosis.^46^, ^47^ This may be the mechanism by which antiviral therapy reduces both overall mortality and CMV related disease in solid organ transplants at 3–18 months post‐transplant.^48^ Secondly, CMV antigens have been directly isolated from smooth muscle cells of the carotid artery and aorta in surgical specimens.^49^, ^50^, ^51^ There is mixed evidence for isolation of CMV antigens or CMV transcripts within the atherosclerotic plaques—Sambiase et al. found no evidence of CMV antigens isolated from cardiac transplantation patients. The presence of CMV within the plaque itself, whilst supportive of a role of CMV in atherosclerosis does not confirm pathogenesis.

**FIGURE 1:**
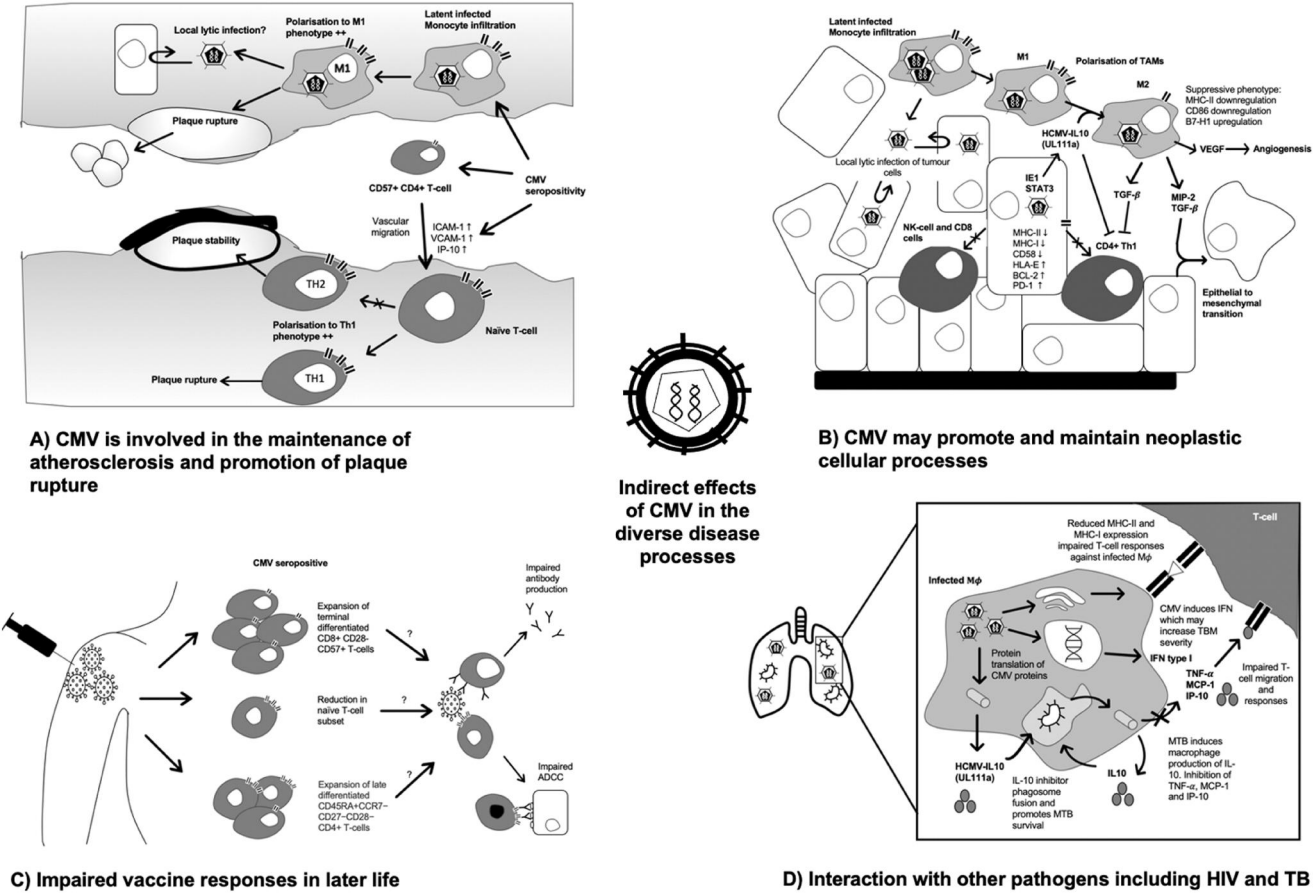
Proposed mechanisms by which cytomegalovirus (CMV) could promote and maintain disease in the general population. (a) CMV likely promotes plaque maintenance through vascular migrating subsets of CD8 T‐cells promoting an inflammatory environment. CMV promotes M1 macrophage and Type 1 T‐helper (Th1) polarisation which are associated with increased risk of plaque rupture. (b) CMV may enter tumours through local infiltration of infected monocytes. CMV has diverse genes which are likely to have oncomodulatory roles. CMV can promote reduced expression of key cancer recognition molecules of cancer cells such as major histocompatibility complex (MHC) and increase inhibitory factors such as PD‐1—therefore impairing immune recognition. (c) CMV has been showed to reduce immune responses—both antibody generation and T‐cell mediated immunity. The mechanism behind this may related expansion of terminally differentiated T‐cell subsets and a reduction of naïve T‐cells therefore reducing ‘immunological space’ for vaccine responses. (d) CMV appears to increase the risk of disease in TB. CMV encodes a molecule similar to IL‐10, whilst TB stimulates production of IL‐10 leading to impaired immune responses local to infection. Both CMV and TB have roles in reduces the expression of MHC receptors and production of chemokines which reduce immune recognition to infected cells.

CMV may initiate and maintain atherosclerosis through multiple immunological mechanisms: promotion of pathogenic T‐cell phenotypes; polarisation of infected monocytes to M1 phenotypes; indirect immune modulation secondary to CMV; and direct lytic damage from reactivation of CMV, potentially originating from invading monocytes (Figure 1a). CMV pre‐disposes to a Type 1 T‐helper (Th1) cytokine phenotype^6^, ^52^ and an M1 macrophage phenotype^19^, ^53^, ^54^ in healthy patients during latency. Type 1 T‐helper cell dominance has been mechanistically linked to the initiation and maintenance of atherosclerosis in mouse models.^55^ Observational studies in humans show that Th1 phenotypes are associated with increased atherosclerosis as measured by carotid artery calcification.^56^ Some studies have shown increased internal carotid interna media thickness in CMV positive patients^7^, ^8^; although, there are other studies which have not shown an association with atherosclerosis on imaging.^6^, ^57^ CMV infection is associated with a subset of vascular‐homing CD57+ CD4+ T Cells^58^ and increased serum levels markers of endothelial activity (ICAM‐1, VCAM‐1, and IP‐10)^52^—indicating a role for CMV in increasing the migration of cells into atherosclerotic plaques (Figure 1a). The risk of atherosclerosis appears to be related to markers of inflammation (C‐reactive protein [CRP], IL‐6)^35^ in combination with raised CMV IgG levels therefore suggesting persistent reactivation as a mechanism.^34^, ^59^

The excess cardiovascular mortality risk may be due to CMV modulating the risk of plaque rupture and subsequent complications (Figure 1a). CD4/CD8 inversion is associated with plaque rupture^60^ and as mentioned, CMV is associated with an inversed CD4/CD8 ratio. Additional co‐infection with other chronic viruses may exacerbate the phenotype; for example, HIV and CMV co‐infection leads to inversed CD4/CD8 ratio and has a particularly high cardiovascular risk.^61^, ^62^ High serum levels of CMV DNA were detected in patients at time of admission with acute coronary syndrome.^63^

## 3 CANCER

The role of CMV in cancer is controversial and the evidence is highly contradictory. The supportive evidence for CMV's role as an oncogenic or onco‐modulatory agent^64^ is twofold; (1) isolation of CMV proteins and DNA from tumours with surrounding non‐infected tissue and (2) well‐studied in vitro effects of CMV on key cellular mechanisms of tumourigenesis—these are briefly summarised below and discussed in more detail elsewhere (Figure 1b).^65^, ^66^

The key mechanisms of CMV tumourigenesis include inhibition of apoptosis, prevention of normal immune surveillance and promotion of cell cycling (Figure 1b). Intermediate‐early 1 (IE1) and intermediate‐early 2 (IE2) are CMV proteins that promote cellular proliferation through suppression of p53 and promotion of entry into the cell cycle. IE2 inhibits apoptosis in infected cells through upregulation of anti‐apoptotic molecules bcl‐2 and c‐FLIP.^67^, ^68^ TGF‐beta is induced by IE1 and contributes to suppression of type‐1 cytokine responses by inhibiting Th1 CD4+ T‐cells.^69^ CMV produces an IL‐10 mimic, which alongside TGF‐beta promotes polarisation of tumour associated macrophages to an M2 phenotype.^69^, ^70^ M2 macrophages are associated with immune evasion of tumour cells, and promote tumour progression through secretion of vascular endothelial growth factor and increasing epithelial to mesenchymal transitions.^70^ Immune recognition of tumour cells by CD4 and CD8 T‐cells is reduced through downregulation of major histocompatibility complex I (MHC I) and MHC II molecules alongside upregulation of PD‐1 signalling. Several CMV proteins promote cell‐cycling such as UL82, which both promotes entry into S phase from quiescence and increases the progress of cells through G_1,_ potentially through an interaction with cell‐cycle regular retinoblastoma protein.^71^ Whilst CMV proteins primarily function to promote viral persistence in the human host, in the appropriate context these may contribute to tumourigenesis.

CMV DNA and protein has been reported within a wide range of tumours—including breast, prostate, glioblastomas—but not the surrounding tissues.^72^, ^73^, ^74^ CMV may come to infect these tissues through local infiltration of infected immune cells with subsequent local infection; however, why tumour cells appear to be susceptible to CMV infection is unclear. Many studies have been unable to isolate CMV DNA or protein in tumours.^40^, ^56^

Glioblastoma is the most studied example of CMV interaction with cancer—particularly in relation to epidemiology, prognosis, and pathogenesis^64^, ^75^—however overall, the evidence is limited. CMV transcripts and proteins have been found within glioblastomas, although this is not a universal finding and there is a large amount of contradictory evidence in this area.^41^, ^74^, ^76^ One study combined several different detection methods including polymerase chain reaction, immunohistochemistry and in situ hybridisation targeted to IE1/2 failed to detect CMV among 125 samples.^40^ Differences between studies is likely due to differences in storage methods, CMV isolation methods and sampling. CMV seropositivity and presence of CMV proteins with tumour has been associated with worse prognosis in several studies.^9^, ^77^, ^78^ However, a recent metanalysis showed no association between CMV seropositivity and prognosis.^39^ Many of the studies included failed to account for confounding factors. A hypothesis‐generating 42‐patient phase I/II double‐blind randomised control trial reported from a post‐hoc analysis a survival benefit in patients with glioblastomas treated with valganciclovir for 6 months compared to patients who had it for less than 6 months (24.1 vs. 13.1 months).^79^ An update of the cohort with 102 patients demonstrated similar findings.^80^ These studies were single centre with a small cohort of patients. Currently there is a multi‐centre double blind randomised phase II trial (NCT04116411) recruiting a cohort of 220 patients with 30 months follow up.

In summary, current evidence demonstrates little substantive proof of a causal interaction between cancer and CMV. The lack of clear causation between CMV and cancer risk may be related to variability in the presence of CMV within tumours and the host response. Alternatively, confounding factors may be driving the associations observed.

## 4 CMV AND SUSCEPTIBILITY TO INFECTION

CMV has been reported to have a protective effect against infection with unrelated pathogens and enhance the response to vaccination and superantigen stimulation.^45^, ^81^ Mice infected with CMV who were challenged with a lethal load of listeria were protected—compared to those with no CMV exposure—and this effect persisted for several months.^81^ Subsequently, the persistence of this effect was questioned as it appeared that the protection waned past 6.5 months in mice.^82^ Protection against unrelated pathogens is often felt to be due to the non‐specific priming of the innate immune system. Alternatively, cross‐reaction between peptides induced in the CMV immune response is another possible mechanism.

TB is a highly prevalent pathogen which has remarkable overlapping epidemiology and cellular tropism to CMV.^16^, ^83^ CMV may increase the risk of progression to disease in TB infected individuals. CMV is nearly ubiquitous in a TB prevalent setting, limiting epidemiological studies between seropositive and seronegative individuals. However, a Ugandan nested case‐control study of 2170 individuals reported increasing levels of CMV IgG are associated with increased risk of TB disease.^42^ This case‐control cohort had age, sex and HIV status matched controls, however there was no control for other confounding factors—particularly adjusting for large family sizes where CMV IgG levels may be raised due to CMV boosting and there is an increased risk of TB. Similarly, in a cohort of South African children, presence of CMV‐specific T‐cell responses as measured by IFN‐y production were associated with a 2.2‐fold increase in progression to TB disease over a 3 year follow up period.^43^

The mechanism of interaction between TB and CMV is unclear.^15^, ^83^ They are both active in similar host‐modulatory pathways including IFN‐y up‐regulation,^52^, ^83^, ^84^, ^85^ IL‐10 upregulation and downregulation of MHC‐I molecules (Figure 1d).^86^ Additionally, CMV is associated with increased CD8+ and CD4+ T cell activation and expansion of CD4+ Human Leukocyte Antigen–DR isotype (HLA‐DR+) T‐cells are associated with an increased risk of progression to TB disease (Figure 1d).^87^

In LMIC settings, CMV and HIV are common co‐infections that are both linked to pro‐inflammatory immune phenotypes. HIV exposure in utero (regardless of whether the infant subsequently develops HIV) appears to increase the risk of CMV infection in infants.^88^ Infants born to a mother living with HIV who themselves are negative for HIV infection (HIV‐exposed, uninfected infants) appear to have adverse outcomes including increased risk of hospitalisation, pneumonia, failure to thrive and higher overall mortality.^13^, ^89^, ^90^, ^91^ In a prospective study of 811 Zambian infants where CMV serostatus was measured at 18‐months, CMV seropositive infants with HIV exposure demonstrated impaired growth and social development scores which persisted compared to CMV‐seronegative HIV‐exposed infants, even when controlling for socioeconomic factors, breastfeeding duration, and education.^14^ CMV‐seropositivity in combination with HIV‐exposure is associated with an increased CRP level in six‐week‐old infants compared to CMV‐seronegative HIV‐exposed infants, which may suggest that CMV and HIV‐exposure are synergistic in promoting an early pro‐inflammatory state in infants.^13^, ^92^ Despite this, CMV and HIV co‐infection does not appear to increase mortality when infants received early anti‐retroviral therapy^14^, ^88^ and the long‐term impact after 2 years of age is unclear.

## 5 CMV AND VACCINATION RESPONSE

CMV may impair response to vaccination—particularly in elderly individuals where both T‐cell and B‐cell mediated effector functions are affected (Figure 1c). In studies evaluating influenza vaccine responses, CMV seropositivity has been associated with poor humoural vaccine response in adult populations.^11^, ^12^ One study demonstrated reduced influenza vaccine IgG production in CMV seropositive adults compared to seronegative adults across all ages,^12^ whereas another found that CMV seropositivity was associated with reduced vaccine response only in adults over the age of 60.^11^ However several studies have shown no association between CMV seropositivity and vaccine responsive to both influenza and pneumococcal vaccination.^10^, ^93^, ^94^ These studies vary in the strain of influenza evaluated and the degree to which they adjust for confounding effects (most studies only adjusted for age and sex).

Various measures of immunogenic response to influenza vaccination—such as neutralising antibodies titres on haemagglutinin inhibition assays and antibody‐dependent‐cellular‐cytotoxicity assays—decrease with age.^95^, ^96^ Poor immunogenic response to influenza vaccination is associated with an increase in the CD8+ CD28− T‐cell subset^96^, ^97^ which is typically expanded in CMV infected elderly individuals as part of a late‐differentiated CD8+ cell phenotype.^20^, ^31^, ^32^ Similarly, late‐differentiated CD4+ phenotypes, present in CMV+ patients, associated with poor humoural immunogenic response in elderly individuals.^11^ In a study evaluating the immune responses to the Ebola vaccines, UK CMV seropositive young adults were found to have reduced antibody production.^98^ Poor response to Ebola vaccination is correlated with increased levels of KLRG1, a marker of the terminal differentiated T‐cell found in CMV seropositive individuals.^98^ The expansion of these differentiated cells is potentially a consequence of low‐level reactivation; it is not known whether these cells contribute directly to poor immune responses or are a proxy marker of an alternative pathological process.

In infants, most of the evidence regarding immunogenic responses to vaccination shows no association between CMV seropositivity and response as measured by vaccine antibodies titres and T‐cell responses. Exposure to CMV occurs early in life in the majority of LMIC's, where seropositivity can reach 90% within 6 weeks of birth.^92^ In a Zambian cohort of 369 CMV‐seropositive infants, there was no difference in polio antibody titres at 18 months of age compared to CMV‐seronegative infants.^44^ In 9‐month‐old Gambian infants, CMV seropositivity was associated with mildly impaired IFN‐y response to measles antigen 1 week after vaccination—however this effect disappeared 3 months later.^45^, ^99^

## 6 CMV RELATED MORTALITY

One interesting aspect of many epidemiological studies is the mortality gap between CMV seropositive and seronegative patients which is not explained by cancer or CVD, even once various socioeconomical factors have been controlled for.^34^, ^35^, ^38^ This non‐specific increase in death is due to a highly heterogenous group of causes.

In a Swedish longitudinal cohort, the accumulation of CD8+ CD28− CD57+ T‐cells, a subset driven by CMV seropositivity as discussed above, was associated with increased mortality (Table 1).^37^ CMV is thought to have a generalised pro‐inflammatory effect potentially through persistent low‐level replication. Raised CRP has been found to be an independent predictor of mortality in the elderly and in another study CRP is synergistic with CMV to increase the risk of mortality.^35^ Overall mortality and CVD‐related mortality was correlated with CMV‐IgG levels and most of this effect was reduced when controlling for IL‐6 and tumour necrosis factor‐alpha levels.^38^ It is likely that CMV interacts with other diseases through modulation of the immune system and localised reactivation in inflammatory niches. Whilst CMV may not initiate disease processes, through biasing immune responses towards pro‐inflammatory phenotypes it may contribute to the maintenance of inflammation and impaired resolution.

## 7 CMV VACCINATION AND THE INDIRECT EFFECTS OF CMV INFECTION

Several CMV vaccine platforms are in phase II and phase III trials including viral vector, recombinant subunit, live attenuated and mRNA.^100^, ^101^ Currently two vaccines have reached phase III. The ASP01131 vaccine recently reported no impact on mortality or CMV end organ damage at 1 year follow up in haematopoietic cell transplant recipients (NCT01877655).^102^ Moderna started recruitment in October 2021 for a phase III trial of an mRNA‐based vaccine which showed promising immunogenicity in phase II trials (NCT05085366). Several different populations are being evaluated in these studies including solid‐organ transplants patients, HSCT patients, adolescent females, women of child bearing age (NCT05085366), and healthy adults. Notably few studies include older adults. Many of these studies include secondary endpoints which evaluate the direct effects of CMV: CMV end organ damage in transplant populations (NCT02506933, NCT01877655) and cases of congenital infection (NCT00125502).

There are no CMV vaccine trials currently evaluating the potential impact on the indirect effects of CMV infection. Registries could be created for any CMV vaccine trial allowing for long‐term follow up and measurement of indirect effects of all participants (Table 2). In addition, using services such as the UK Clinical Practice Research Data Link (CPRD) for data linkage studies would allow follow up of long‐term outcomes of patients with a rich dataset that includes both primary care and secondary care data. Many previous phase II or III trials^102^, ^103^, ^104^, ^105^, ^106^ have been conducted in a healthy adult or child population with no existing co‐morbidity—but little long‐term follow up of these patients exists. Only the Moderna mRNA‐1647 vaccine (NCT04975893) currently has registered trial for longer follow up of participants. Bernstein et al. (NCT00133497 and NCT03486834) included adolescent female and child‐bearing age females respectively—these are healthy females that have comparable demographics to the general population—follow up of cohorts such as these could provide data broadly applicable to the general population in high income country settings. Registries including all clinical trial patients would allow for comparison between the unvaccinated and vaccinated patients within the same trial, and comparison between different vaccine candidates. Several of the indirect effects of CMV discussed above will not be detectable within conventional vaccine trial follow up periods, as these effects are likely to take decades to emerge; therefore, it is important to collect longitudinal data through registries or data linkage.

**TABLE 2:** Proposed areas of research to better understand the impact of a cytomegalovirus (CMV) vaccine

Indirect effect knowledge gap		Study design(s)	Outcome measures
TB/HIV	Pathogenesis—role of reactivation of CMV in driving TB	Prospective birth cohort study measuring CMV viraemia during active TB infection. Focussed on LMIC settings	Presence of CMV Viraemia during active TB disease; cytokine measurements; transcriptome and proteome
		Mouse models with CMV latent infection with TB challenge	
	Epidemiology	Prospective cohort birth studies. Measurement of CMV serostatus of mother during pregnancy. Sequential measurement of CMV serostatus of baby. Ideally measure CMV viraemia	Incidence of TB disease in CMV seropositive versus seronegative
			Rate of progression from TB infection to TB disease in CMV seropositive versus seronegative.
			Role of anti‐viral therapy in prevention of TB disease or anti‐viral therapy host‐modulation in TB disease
	Measuring vaccine impact	CMV vaccination trial registries facilitating long‐term prospective follow up;	Rate of progression from TB infection to TB disease in vaccination compared to un‐vaccinated
			Incidence of TB disease in vaccinated compared to general population
		Modelling studies evaluating the impact of vaccination on TB disease outcomes. Should include different populations and include LMIC population demographics	Incidence of site of TB disease (rate of pulmonary vs. extrapulmonary TB) in vaccinated compared to unvaccinated (or general population)
			Correlates of protection against TB
		Evaluation of effectiveness of BCG vaccination—prospective follow up of paediatric population post‐BCG vaccination	Number and severity of TB cases
			Measurement of immune correlates of protection post‐BCG vaccination
Infectious disease interactions	Epidemiology	Prospective cohort study evaluating rate of reactivation of CMV in different contexts including ICU versus ward and illnesses	Rate of CMV viraemia in different group (ICU vs. ward, pneumonia vs. UTI)
			Mortality and mortality rates in CMV viraemia versus no CMV viraemia
	Vaccination impact	Modelling study evaluating the excess deaths and morbidity cause by CMV reactivation in severe illness	Measurement of excess deaths secondary to CMV reactivation in severe illness
CMV influence on vaccination response	Vaccination impact	CMV vaccination trial registries facilitating long‐term prospective follow up;	Measurement of influenza (or alterative) vaccine IgG/HA assay titres and ADCC at various time points in vaccine recipients
		Data linkage studies	Rates of admission to hospital with vaccine preventable diseases (influenza, streptococcal pneumonia)
		CMV vaccine trials. Prospective data during early follow up	Measurement of influenza (or alterative) vaccine IgG/HA assay titres and ADCC in infants born to mother who have had vaccination
Atherosclerosis and CVD risk	Pathogenesis	Autopsy samples from ACS patients with CMV seropositivity and CMV seronegative.	Presence of CMV inclusion bodies. Presence of CMV DNA
		Pro‐atherosclerosis mouse studies	M1/M2 macrophage polarisation and RNA transcriptome
	Epidemiology	Prospective cohort studies of initially CMV seronegative population. To include measurement of serostatus sequentially; measurement of CMV viraemia; frequency of reactivations and measurement of socioeconomical cofounders	Incidence of acute coronary syndrome (ACS)
			Coronary artery thickness
			Incidence of peripheral vascular disease
			Biomarkers predictive of outcomes above
	Measuring vaccine impact	CMV vaccination trial registries facilitating long‐term prospective follow up;	Incidence of acute coronary syndrome (ACS)
			Coronary artery thickness measurement
		CMV vaccination trial follow up: Measurements including rates of CMV viraemia; CD4/CD8 ratio	Incidence of peripheral vascular disease
			Correlates of protection against atherosclerosis following vaccination
Non‐specific deaths	Measuring vaccine impact	CMV vaccination trial registries facilitating long‐term prospective follow up;	Non‐specific mortality rate in CMV
		CMV vaccination trial follow up. To include measurements of CMV impact such as rates of CMV viraemia; CD4/CD8 ratio	Correlates of protection against non‐specific mortality

Abbreviations: BCG, Bacillus Calmette‐Guerin; HA, Haemagglutination; UTI, Urinary tract infection.

Currently, vaccine modelling and cost effectiveness studies have only considered the direct effects of CMV.^107^, ^108^, ^109^ Modelling the indirect effects of CMV is severely limited by lack of detailed epidemiological evidence which can be used to inform accurate parameters (Table 2). There are several well defined long‐term prospective cohort studies with CMV seropositivity data available—NONA, OCTA, EPIC (Table 1)—which could be used to form initial estimates of the impact of vaccination in reducing the burden of atherosclerosis and CVD. Using data from previous studies demonstrating increased mortality from CMV reactivation in intensive care unit, an estimate of excess deaths caused by CMV reactivation in ICU‐admitted pneumonia patients is also feasible. Future studies need to evaluate the epidemiological role and quantitative impact of CMV on outcomes such as CVD, TB, HIV, vaccination responses and cancer (Table 2), which will allow for more accurate modelling studies to be performed and estimate the impact of CMV vaccination. During the COVID‐19 pandemic, several HICs used population based observational data to inform the effectiveness of societal lockdowns, vaccine effectives and other public health strategies. Researchers could adopt these platforms, utilise existing routinely collected national datasets and develop novel strategies using anonymised electronic healthcare records to conduct observational studies evaluating the association between CMV and the conditions outlined.

It is important to note that there is evidence that latent CMV infection may have beneficial effects, particularly in children and young adults. Seropositive 20–30‐year‐olds appear to have a greater response to influenza vaccination compared to seronegative individuals of the same age at 28 days post vaccination.^94^ Additionally, in Gambian infants IFN‐y response to CMV correlated with IFN‐y response to staphylococcal enterotoxin B (*r* = 0.30, *P* = 0.012). In young adults CMV was associated with high IFN‐y, pSTAT1 and pSTAT3 level suggestive of an activated immune state. In younger individuals, it is plausible that CMV may promote a more dynamic active immune system, and therefore vaccination may be harmful.

All current modelling and cost‐effectiveness studies are derived from data in HICs where seroprevalence is lower and seroconversion typically occurs later in life. Limited epidemiological data in many LMIC countries hinders detailed modelling in these settings. In LMIC settings, the indirect effects of CMV on infectious disease outcomes—where TB and HIV prevalence is high—are likely to be an important element of modelling the cost‐effectiveness of a vaccine. Further modelling studies in this population should evaluate outcomes such as HIV and TB reactivation and mortality, and model how outcomes vary between different vaccination target populations.

## 8 CONCLUSIONS AND IMPLICATIONS FOR VACCINE DEVELOPMENT

Whilst studies have modelled how CMV vaccination would impact the reduction of CMV associated disease in immunosuppressed patient and congenital disease, there may be unforeseen benefits of vaccination in the general population. Atherosclerosis and CVD have been associated with increased CMV IgG levels and there are clear mechanisms by which CMV could maintain atherosclerosis and promote plaque rupture (Table 1). Vaccination responses in CMV positive individuals, whilst unaffected in infancy, may be impaired in later life as demonstrated by poorer response to influenza vaccine. Additionally, CMV may synergise with other important pathogens such as TB where there is an increased risk of TB progression and disease. Many of these effects may be mediated by recurrent sub‐clinical CMV reactivation which leads to immune activation and expansion of dysfunction immune cell subsets. Following participants of vaccine studies may be the most effective way to evaluate the effect of CMV in the general population and there should be consideration for creation of registries in these trials.

## AUTHOR CONTRIBUTIONS

Seilesh Kadambari conceived the idea for the paper. Philip Moseley wrote the first draft of the manuscript. Philip Moseley, Paul Klenerman and Seilesh Kadambari contributed to the literature review and provided scientific content to each section of the manuscript.

## CONFLICT OF INTEREST

No conflict of interest declared.

## Data Availability

Data sharing not applicable to this article as no datasets were generated or analysed during the current study.
